# Effect of ointment-based egg white on healing of second- degree wound in burn patients: a triple-blind randomized clinical trial study 

**Published:** 2019

**Authors:** Simin Jahani, Hadis Ashrafizadeh, Kamran Babai, Amir Siahpoosh, Bahman Cheraghian

**Affiliations:** 1 *Nursing Care Research Center in Chronic Diseases, Nursing and Midwifery School, Ahvaz Jundishapur University of Medical Sciences, Ahvaz, Iran.*; 2 *Nursing and Midwifery School, Student Research Committee, Ahvaz Jundishapur University of Medical Sciences, Ahvaz, Iran.*; 3 *Department of Plastic Surgery, Taleghani Hospital, Ahvaz Jundishapur University of Medical Sciences, Ahvaz, Iran. *; 4 *Medical Plants Research Center and Department of Pharmacology, School of Pharmacy, Jundishapur University of Medical Sciences, Ahvaz, Iran.*; 5 *Department of Biostatistics and Epidemiology, School of Public Health, Ahvaz Jundishapur University of Medical Sciences, Ahvaz, Iran.*

**Keywords:** Burn, Egg white, Traditional medicine, Silver sulfadiazine, Patient

## Abstract

**Objective:**

Burn wound healing is one of the problems of medical sciences and it is of great importance to find a drug or substance that can heal burn wounds with minimum complications. The present study aimed to evaluate the effect of ointment-based egg white on healing second-degree burn wounds.

**Materials and Methods:**

In the present triple-blind clinical trial, a total of 90 patients from Taleghani hospital, Ahvaz, Iran were selected and randomly divided into two groups based on the inclusion criteria. The intervention group was dressed with egg white formulation + silver sulfadiazine cream and the control group was treated with placebo + silver sulfadiazine cream. The burn wound healing process was evaluated on days 1, 7 and 15 by the Bates-Jensen wound assessment tool.

**Results:**

The mean scores of wound healing were decreased (13.75±1.83) in the intervention group when compared to the control (21.51±5.7) on day 15 (p<0.001). The mean duration of wound healing, wound depth, edges, undermining, necrotic tissue, amount of necrosis, exudate type and amount, surrounding skin color, wound induration, peripheral edema, granulation, and epithelialization were significantly decrease in intervention group in comparison with control (p<0.001).

**Conclusion:**

The findings of this research showed that egg whites formulation is an appropriate treatment for burn wound healing, reduced above-noted burn wounds’ variables. It seems that this treatment, along with the common medicine, improves chronic wound recovery rate and patients’ health status.

## Introduction

Burn wound is an injury associated with skin damage, which along with deep wound area negatively affects the person, family, and society. The burned people who were treated for a long time, suffer from superficial abnormalities (Bunyan, 1983 ▶). Burns account for 5 to 12% of all traumas in the world, and have been assigned the fourth place of the most common traumas after accidents, crashes, and interpersonal violence, respectively (Arthur, 2014 ▶; Mathers, Fat, and Boerma, 2008 ▶). The WHO reported that more than 300,000 people die every year due to burn injuries, 95% of whom being from low and middle-income countries (Organization, 2002). So, it is of great importance to find a medicine or substance that can heal burn wound with minimum complications (Brunner, Smeltzer, Bare, Hinkle, and Cheever, 2010 ▶). Among these treatments, silver sulfadiazine 1%, as a common burn healing treatment is widely used in patients. Silver sulfadiazine, as a sulfonamide-based topical agent, has antibacterial and antifungal effects that is topically used in partial thickness and full thickness burns to prevent infection. Dressing with silver sulfadiazine produces a false scar because of its adhesion to the wound surface and it’s a toxic effect on the repair of keratinocytes that induces a delay in wound healing process (Dunn and Edwards-Jones, 2004 ▶; Wasiak, Cleland, and Campbell, 2008 ▶). This ointment has several complications such as leukopenia, methemoglobinemia, silver poisoning, and skin discoloration (Sood and Achauer, 2006 ▶). Other drugs that are used to repair burn wound are nitrofurazone, mafenide acetate, and corticosteroids (Brunner et al., 2010 ▶). The use of supplementary, traditional, and complementary therapies, as low-risk, cost-effective, easy-to-use methods with limited side-effects is becoming widespread in different countries (Harlow, 2013 ▶). A review study showed that several plants such as garlic, *Aloe vera*, coriander, and spoon juice produce better results when used for wound healing than silver sulfadiazine (Bahramsoltani, Farzaei, and Rahimi, 2014 ▶). Eggs are one of the animal products that have been noted in traditional medicine (Hasanzadeh and Mehdikhanloo, 1983 ▶). The egg white protein is easily absorbed into the muscle. Each pure egg white (without yolk) contains about 3.6 g pure protein including all the essential amino acids and vitamins required for the body (Kelly, Plat, Haenen, Kijlstra, and Berendschot, 2014 ▶). Eggs have therapeutic, immunogenic and functional properties in addition to high nutritional value (Fernandez, 2010 ▶). The presence of lysozyme, G2 and G3 globulins, and ova macroglobulin, immunoglobulin Y, and other antimicrobial compounds in eggs could induce immunogenicity and antimicrobial properties (Kelly et al., 2014 ▶). Tryptophan is a vital compound and essential amino acid which is present in egg white. The topical application of this amino acid could improve burn wound healing in mice by increasing re-epithelialization, cell proliferation and neovascularization process (Alia Sadiq, 2018 ▶). Further, a similar effect was observed in riboflavin-administered rats (Lakshmi, Lakshmi, and Bamji, 1989 ▶). Hassanzadeh et al. using a rat model, showed that the rate of burn wound healing variables including inflammation, edema, and hemorrhage in the group receiving the extract of cabbage and egg whites was better than other groups (Hasanzadeh and Mehdikhanloo, 1983 ▶). Nurses are the first caregivers who are responsible for cleansing debris, and daily observation of the wound. Therefore, they play an essential role in selection and evaluation of the outcome of wound care (Darvishpour, Lotfi, Salehi, Aghazadeh, and Aali, 2007 ▶). Hence, it is imperative for nurses to update their knowledge through the latest guidelines and learn new methods of treatment to provide effective services and promote health status in patients (Aalaa, Malazy, Sanjari, Peimani, and Mohajeri-Tehrani, 2012 ▶). Chronic wound is the costliest unsolved health problems. Currently, treating chronic wound involves wound healing, antibiotic treatment, and sometimes removal of damaged tissues by surgical techniques. A large number of patients suffers from chronic wound (Thomas, Goode, LaMaster, and Tennyson, 1998 ▶). Considering, the rich content of egg whites, as mentioned above, and their effects on wound healing, as well as growing interest of people in the use of natural-derived treatments (Hasanzadeh, Nouri, Hajiabadi, Soltan, and Javadi, 2005 ▶), and limited evidence for the effects of egg whites on burn wound healing, the present study aimed to investigate the effect of ointment-based egg white on second-degree burn wound healing in patients referred to Taleghani hospital, Ahvaz, Iran. 

## Materials and Methods

**Sample size**

The present triple-blind clinical trial (No: U-96031) was conducted in Taleghani hospital, Ahvaz, Iran, in 2017. Researchers, patients, and analysts were blind. In order to determine the sample size, comparison of ratios formula was used and α=0.1, β=0.2, p1=0.32 and p2=0.06 were considered. Based on the calculated sample size, 2 groups of 42 people (total of n=84 individuals) were designed (Hasanzadeh and Mehdikhanloo, 1983 ▶). Eventually, considering a dropout rate of 0.5%, 45 people were enrolled into each group (a total of 90 individuals). Subjects were allocated to each treatment groups by blocked randomization and using 6 blocks.

$n=\frac{{\left(Z_{1-\frac{a}{2}}+Z_{1-\beta }\right)}^{2}p_{1(1-p_{1})}+p_{2(1-p_{2})}}{{\left(p_{1-p_{2}}\right)}^{2}}$

**Patients**

The inclusion criteria were having a second-degree burn wound confirmed by the physician, being of 18-65 years old, a burn percentage of <15%, arriving at the burn center within 6hr post burn injury, not having facial, genital, and perineal burns (due to the sensitivity of these areas), having normal hemoglobin, and total protein levels, lack of wound contamination, receiving no prior treatment on the wound except for drinking water, absence of symptomatic infections on parts of the body, and not having underlying immune deficiency diseases or skin allergies. The exclusion criteria included being in need for receiving immunosuppressive drugs, steroids, chemotherapeutics, symptoms of infection in the burn wound, and radiotherapy during the study. In order to observe ethical considerations, after receiving the ethical code IR.AJUMS.REC.1396.146, the researcher explained the purpose of the research, the research method and its safety, the freedom to participate in the study, and the preparation of egg white ointment formulation in the laboratory and administration of that in animal models under similar conditions of patients with burn wound injury. To produce topical formulation containing egg whites, eggs were freshly prepared from aviculture and kept refrigerated until used. A topical formulation was prepared and the required tests to confirm the formulation were performed. Then, the formulation was studied clinically. The placebo was comprised of cream compounds of the treatment group but lacked egg whites. These products were produced by the pharmacologists at Medicinal Plants and Drug Research Institute of Ahvaz Jundishapur University of Medical Sciences, Ahvaz, Iran. In this study, superficial and deep second-degree burn wound were studied and none of the patients were deprived of their treatment with silver sulfadiazine 1% cream. These patients referred to the hospital as an out-patient and were assessed. They were referred to the emergency department of the hospital during each day of the first week and, depending on the recovery rate, in the second week. 

**Study treatment**

The treatment protocol was as follows: The wound site was exposed to the air for 5 min after washing with normal saline in both cases of superficial second-degree burn wound with full blisters, and deep second-degree burn wound with destroyed blisters and removed blisters and tissues until be dried. Then according to the wound size, the egg white ointment was applied to the wound site by sterile tongue depressors. Next, silver sulfadiazine 1% cream was placed on the egg white ointment and the wound was dressed. The above steps were repeated for the control group, which was treated with placebo. In order to control allergic complications induced by egg white, patch testing was performed for each patient. In this study, a demographic questionnaire consisting of 11 questions (date and time of burn occurrence, current weight of the patient, date and time of referral, age, gender, educational level, average family income, occupation, burn percentage, and location and depth of burn) was used. The burn site and percentage were determined according to the Lund and Browder chart. Bates-Jensen wound assessment tool has a 15-item questionnaire; although two statements of questionnaire about wound location and form are not categorizing, the other 13 statements are scored according to the 5-point Likert’s scale in which point 1 indicates the best status and point 5 is the worst situation. The minimum and the maximum scores are 13 and 60, respectively. Lower scores suggest better wound healing. These statements deal with the wound size, depth, edges, and undermining, necrotic tissue type, amount of necrotic tissue, granulation and epithelialization tissue, exudate type and amount, surrounding skin color, edema, and induration. The wound shape is examined based on the wound pattern, length, width and depth. Aplastic tape meter instrument which has a precision of 1:1000 was used to measure wounds. At the time of admission to the clinic (i.e. the first day) and on the seventh and fifteenth days, the wound extent was measured by the tape meter instrument and expressed as length×width. The scientific validity of Bates-Jensen wound assessment tool was calculated using face and content validity method. Content validity index (CVI) in this research was 89% with p=0.05. Inter-rater reliability that was measured to check the scientific reliability of the data collection tool, was 0.86 among observers. Also, since the Bates-Jensen wound assessment tool is a Likert-based tool and has an inter-rater agreement value of 0.20 for each item, the kappa coefficient was measured and found to be above 60% for each one. Moreover, since dressing method and skill of individuals affect wound healing, the researcher was well trained in measuring the wound and using the tools. Digital images were taken from patient’s wound during the treatment stages, and the third observer who was blind to the dressing method, investigated the process of wound healing. Written consent form was obtained from all patients. The researcher evaluated the observed content soon after observation in order to avoid any contradiction and ambiguity caused by the interval between observation and study. 

**Statistical analysis**

Data analysis was performed using SPSS v. XVI and presented as mean±standard deviation (SD); the results of the two groups were compared using repeated-measures analysis of variance (ANOVA), followed by Chi-squared and Fisher’s post hoc tests. A p<0.05 was considered statistically significant.

## Results

**Patient’s characteristics**

As shown in Table 1, there were no remarkable differences between the two groups in terms of age, gender distribution, income status, educational level, and occupation. According to the Table 2, there was no significant difference between the control and intervention groups regarding the mean burn percentage, shape, location, and depth before initiation of the treatment. 

**Table 1:** Comparison of demographic characteristic between the studied groups

Demographic variables		control (n=45)		intervention (n=45)		Total		*p*-value
		n	%	n	%	N	%	
Age*	18-30	16	35	18	40	34	37	0.78
	31-40	20	44	13	28	33	36	
	41-50	5	11	6	13	11	12	
	51-65	4	8	8	17	12	13	
Gender+	Male	23	51	18	40	41	45	0.4
	Female	22	48	27	60	49	54	
Educational level**	Elementary	13	28	15	33	28	31	0.071
	Middle school	3	6	10	22	13	14	
	Diploma	25	55	15	33	40	44	
	Bachelor and higher	4	8	5	11	9	9	
Economic level**	Under one million	4	8	14	31	18	20	0.099
	Between one to three million	36	80	27	60	63	70	
	Above three million	5	11	4	8	9	10	
Occupational+	Unemployed	9	20	9	20	18	20	0.8
	Employee	14	31	10	22	24	26	
	Free	8	17	9	20	17	18	
	Housewife	14	31	17	37	31	34	

Data are presented as number (%)

*: Independent T-Test,

+ : Chi-square Test,

**: Fisher tests.

**Table 2:** Comparison of characteristics burn wound between the studied groups

Characteristics wound		control (n=45)		intervention (n=45)		Total		*p*-value
		n	%	n	%	n	%	
Burn percentage*	Between 0-3	30	69	31	67	61	66	0.59
	Between 3-6	10	21	12	25	22	23	
	Between 6-12	5	10	2	4	7	14	
Burn depth+	Second degree superficial	38	84	35	77	73	81	0.6
	Second degree depth	7	15	10	22	18	18	
Location of burn**	Upper member	27	60	28	62	55	61	0.56
	Lower member	18	40	17	37	35	38	
Shape of burn**	Irregular	6	13	9	20	15	16	0.68
	Round and oval	24	53	20	44	44	48	
	Linear	3	6	6	13	9	10	
	Screw and crook	6	13	6	13	12	13	
	Other forms	6	13	4	8	10	10	

Data are presented as number (%)

* : Independent T-Test,

+ : Chi-square Test,

** : Fisher tests.

**Effects of egg white ointment on burn wound variables**

The variance of wound size between the intervention and control groups was not homogeneous based on the Mauchly’s test (p<0.05). So, there was no assumption of compound symmetry. Generally, wound improvement was achieved based on the intragroup Greenhouse-Geisser test, in both groups (p<0.001). Comparing wound size results by repeated measures ANOVA test revealed no significant difference between the intervention and control groups. The Mauchly’s test showed that the variance between the two groups was homogeneous in terms of wound depth, undermining of underlying tissues edges, exudate type, skin color around the wound, induration, and peripheral edema. Thus, the assumption of compound symmetry was established, and the wound recovery rate was achieved based on the intragroup Greenhouse-Geisser test in both groups (p<0.001). The mean duration of wound healing in intervention group was significantly lower than that in the control based on the repeated measures ANOVA test (p<0.001). The results of Mauchly’s test showed that the variances between the two groups were not homogeneous in examining the wound edges, necrotic tissue type, exudate amount, granulation, epithelialization (p<0.05), and amount of necrotic tissue (p<0.001). Therefore, assumption of compound symmetry was not established. Further, based on the intragroup Greenhouse-Geisser test, wound recovery rate was achieved in both groups over time (p<0.001). 

The repeated measure ANOVA results indicated that the mean duration of wound healing in intervention group was significantly lower than that in the control (p<0.001; Table 3). The mean scores of wound healing were decreased (13.75±1.83) in the intervention group when compared to the control (21.51±5.7) on the fifteenth day (p<0.001). Table 4 presents the average percentage of wound healing at three time points for the studied groups. 

**Table 3:** Variables related to the burn wound between the studied groups

Bates-Jensen wound assessment tool		1st Day		7th Day		15th Day		*p*-value+
		mean	SD*	mean	SD	mean	SD	
Wound size	Control group	2	0.9	2	0.78	1	0.86	0.32
	Intervention group	2	1.08	2	1.03	1	0.75	
Wound depth	Control group	2	0.28	2	0.48	1	0.53	<0.001
	Intervention group	2	0.42	1	0.48	1	0.14	
Wound edges	Control group	2	0.69	2	0.39	1	0.60	<0.001
	Intervention group	2	0.66	1	0.50	1	0.25	
Undermining	Control group	2	0.53	2	0.57	1	0.61	<0.001
	Intervention group	2	0.79	1	0.58	1	0.14	
Necrotic tissue type	Control group	2	0.43	2	0.68	1	0.54	<0.001
	Intervention group	2	0.79	1	0.57	1	0.29	
Amount of necrosis	Control group	2	0.62	1	0.48	1	0.53	<0.001
	Intervention group	2	0.78	1	0.51	1	0.14	
Exudate type	Control group	3	0.47	2	0.95	1	0.81	<0.001
	Intervention group	3	0.71	1	0.70	1	0	
Amount of exudate	Control group	3	0.66	2	0.67	1	0.58	<0.001
	Intervention group	3	0.96	1	0.66	1	0	
Surrounding skin color	Control group	3	0.86	2	0.72	1	0.78	<0.001
	Intervention group	2	0.98	1	0.52	1	0.14	
Induration	Control group	2	0.68	2	0.66	1	0.70	<0.001
	Intervention group	2	0.96	1	0.52	1	0.14	
Peripheral edema	Control group	2	0.63	1	0.56	1	0.61	<0.001
	Intervention group	3	1.12	1	0.48	1	0.29	
Granulation tissue	Control group	4	0.20	3	0.70	1	0.80	<0.001
	Intervention group	4	0.55	1	0.57	1	0.20	
Epithelialization tissue	Control group	5	0	3	0.67	1	0.71	<0.001
	Intervention group	4	0.50	1	0.50	1	0.14	

Data are presented as mean ± SD; n= 45

+ : repeated measures ANOVA Test, SD

* : Standard deviation.

**Table 4:** Comparison of mean wound healing in three time intervals between the studied groups

mean wound healing	1^st^ Day		7^th^ Day		15^th^ Day		*p*-value*
	mean	SD	mean	SD	mean	SD	
Control group	42.04	4.1	31.42	4.8	21.51	5.7	<0.001
Intervention group	41.46	6.6	19.73	5.42	13.75	1.83	

Data are presented as mean ± SD; n= 45

+ : repeated measures ANOVA Test.

**Figure 1:**
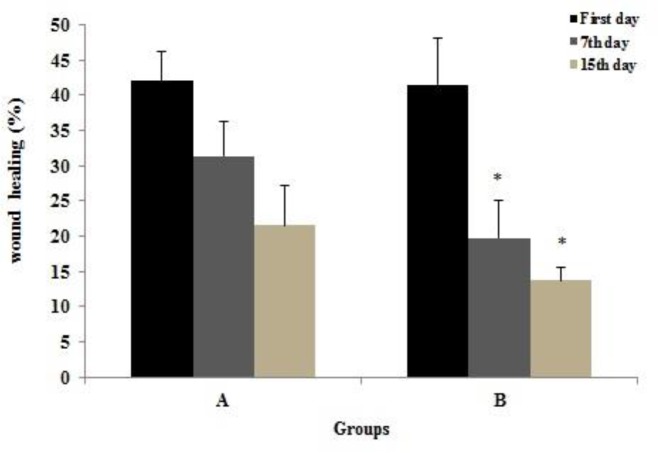
Comparison of mean wound healing percentage in three time intervals between the studied groups. Data are presented as mean ± SD; n= 45; ^*^p<0.001 Compared to control group. (A) Control; (B) intervention

According to the intragroup Greenhouse- Geisser test, wound recovery percentage was decreased in both groups on the seventh and fifteenth days. Also, this variable revealed similar alterations when compared by the repeated measures ANOVA test between the intervention and control groups (p<0.001; Figure 1). 

**Figure 2:**
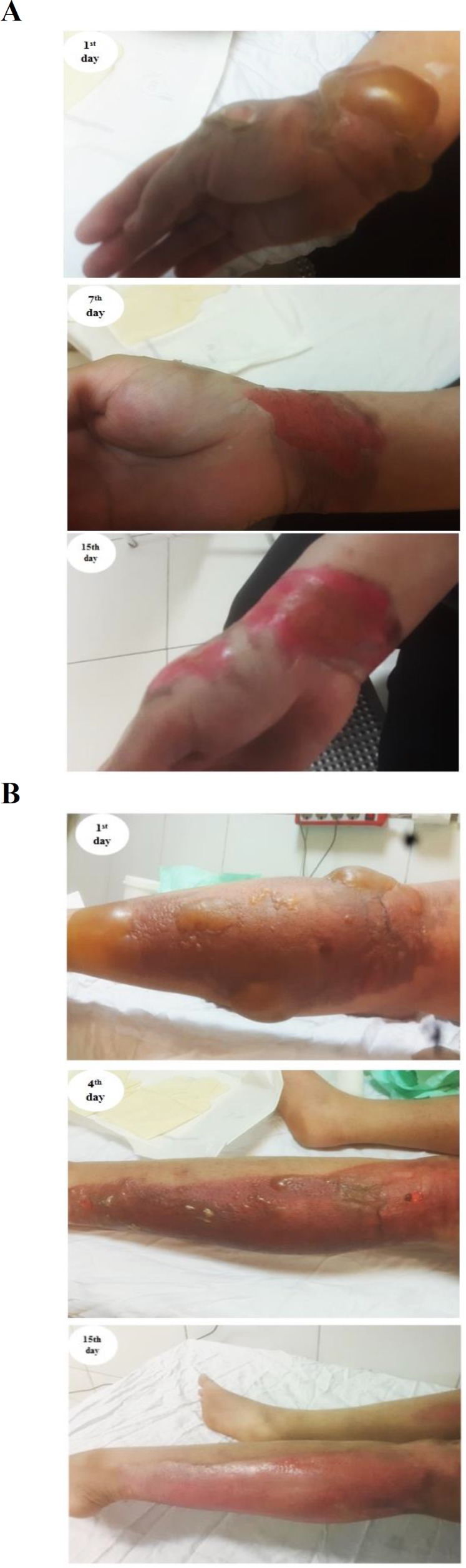
Comparison of treatment time in three time intervals between the studied groups. (A) Control; (B) intervention

Also, comparison of the duration of second-degree burn wound healing showed that most of the patients treated with egg white ointment in addition to silver sulfadiazine 1% in the intervention group showed improvement from day 5 to 10, while the patients treated with placebo in addition to silver sulfadiazine 1% in the control group recovered from day 10 to 17. So, a significant difference was observed between the intervention and control groups in terms of this variable (p<0.001; Figure 2). Importantly, there was no post-treatment complication in the intervention group, but 5 cases of control group were referred to the emergency department of the hospital with allergic complications such as itching, redness, and hives after one week of burning treatment, and treated with corticosteroids.

## Discussion

The results of the present study showed that egg white has a marked effect on the treatment of deep and superficial second-degree burn wound. However, the wound’s shape was improved in both groups over time and there was no significant difference between the groups in this regard. Consistent with the present study, Hassanzadeh et al. showed that the dermis in the groups treated with cabbage leaves extract and egg whites was similar to the that of silver sulfadiazine-treated group, and the density of fibroblasts was increased in the burn area (Hasanzadeh and Mehdikhanloo, 1983 ▶).

In the present work, the wound size did not improve during the treatment period in intervention groups, but the wound depth and edges were recovered in egg white-treated patients. Further, necrotic tissue type, necrotic tissue level, type and amount of exudate, peripheral edema, and induration recovered in intervention group on days 1, 7, and 15. Hassanzadeh et al. (2005) ▶ revealed that the egg yolk eliminates the edema, inflammation, and red blood cells from the wound surface (Hasanzadeh et al., 2005 ▶). Also, Azarpira et al. reported decreased inflammation in the burn area, but very little inflammation was observed in the peripheral arterial regions (Rastegar F, 2011 ▶). So, our results are in accordance with the above-mentioned studies. 

The present study demonstrated that the red color around the wound, granulation, and epithelialization were decreased in egg whites ointment-treated patients on days 1, 7, and 15. Hassanzadeh et al. showed that utilization of red cabbage extract and egg whites completely restored the epidermis, stratum corneum, and the wound’s scar and induced hair growing in treated animals (Hasanzadeh and Mehdikhanloo, 1983 ▶). Moreover, the results of Azarpira et al., study showed that epithelialization was performed without any evidence of scaling or scar diagnosis in egg yolk-treated group (Rastegar F, 2011 ▶). 

The present results indicated that the healing period was shorter in the intervention group. Consistent with the present results, Imeni et al. (2010) reported that the duration of second-degree burn wound healing in the experimental group dressed with tragacanth gel was 7 to 10 days while 11 to 14 days in control group that received sulfadiazine ointment (Imani, Kkermanshahi, and MoosaviI, 2010 ▶). In our research, the percentage of wound healing on days 1, 7, and 15 showed significant differences between the control and intervention groups. This result is in accordance with the Hassanzadeh et al. (1983) ▶ study that revealed that the mixture of red cabbage and egg whites has a significant effect on the healing of second-degree burn wound, with faster repair processing, 100% recovery or fewer scars in treated group (Hasanzadeh and Mehdikhanloo, 1983 ▶). Another study conducted by Hassanzadeh et al. (2005) ▶ indicated that wound healing rate had better status in groups treated with egg yolk and silver sulfadiazine than the negative control group which are consistent with the results of the present study (Hasanzadeh et al., 2005 ▶). In general, egg whites can be effective in repairing second-degree burn wound. Previous studies showed that egg whites induce a positive effect on the recovery of burn wound via increasing blood flow and reducing inflammation or infection (Hasanzadeh and Mehdikhanloo, 1983 ▶). Intracellular and extracellular content of glutathione present in egg whites play an important role in cell resistance to poisoning or burn (Hasanzadeh et al., 2005 ▶). In addition, it was revealed that the effects of antioxidants including vitamins A, E, and C and trace elements such as zinc, copper, and selenium accelerated wound healing. Egg whites contain these compounds and certain dose of them is effective in burn wound healing (Bang and Dashti, 1995 ▶; Canapp et al., 2003 ▶; Chai, Guo, and Sheng, 1995 ▶; Dreizen, 1979 ▶; Ianev, Radev, Balutsov, Klouchek, and Popov, 1995 ▶; Mathus-Vliegen, 2004 ▶; Morley and Silver, 1995 ▶; Sieradzki et al., 1998 ▶). Some physicians suggest that spraying vitamin E capsules on the burned can also improve wound healing (van Henegouwen, Junginger, and de Vries, 1995 ▶). Therefore, it could be suggested that egg white-therapeutic effects on burn wound are mediated by Vitamin E and above-noted elements. Previously, it was shown that L-Argin present in egg leads to reduced wound inflammation, increased speed of the necrotic tissue cleansing, accelerated proliferation of epithelial cells, and wound healing. So, this amino acid reduce glucose levels, and increase the growth factor in the wound (Ge et al., 2003 ▶). The rate of wound healing has vital effects on the patient’s life. For example, the treatment time duration decreases by applying epidermal growth factor and accelerating the growth rate of the skin area in patients with severe burn removing injured skin from the area. Covering the burn wound with the patient own skin reduces the risk of subsequent infection, rejection, mortality, and hospitalization time (Knighton, Ciresi, Fiegel, Austin, and Butler, 1986 ▶). Therefore, in agreement with previous research, in the present study, reduced hospitalization time, infection, and costs in burn patients were observed. 

The findings of the present study showed that egg whites formulation is an appropriate ointment for burn wound. This ointment induced its effects by reducing the mean duration of wound healing, depth, edges, undermining, necrotic tissue, amount of necrosis, exudate type and amount, surrounding skin color, induration, peripheral edema, granulation, and epithelialization tissues in treated burn patients. Owing to the importance of reviving traditional medicine and its lower side effects, it seems that the use of this compound, along with other common medicines, improves the chronic wound and promotes the health status at the individuals and societal levels. As a direction for future studies, it is recommended to investigate the effects of egg whites on deep-superficial second-degree burn wound and other ulcers such as diabetic foot and bedsore.

One of the research limitations was collecting the samples from outpatients, because there was a problem of lack of control over the research subjects during 24 hours. To resolve this problem, at the beginning of the study, the patients or their companions were trained on how to care for the dressings, and they were given a phone number for the follow-ups. Wound healing depends on a number of factors including characteristics of blood flow, the patient’s nutrition, immune system, etc. Therefore, the patient’s system of defense and nutrition can affect the speed of wound healing. The differences in patients’ economic status and nutrition can affect the speed of burn wound healing. The researchers recommend consumption of high-calorie and high-protein foods, vegetables, and fruits while highlighting the importance of proper nutrition and introducing the foods needed to heal patients.
